# Relationship between triglyceride-glucose index baselines and trajectories with incident cardiovascular diseases in the elderly population

**DOI:** 10.1186/s12933-023-02100-2

**Published:** 2024-01-03

**Authors:** Yue Wang, Xueyu Chen, Jie Shi, Mingyi Du, Shengnan Li, Jinhong Pang, Junpeng Qiao, Yingying Zhao, Qiaoqiao Chen, Yuanyuan Guo, Yan Xi, Weiwei Chi

**Affiliations:** 1 Department of Epidemiology, School of Public Health, Cheeloo College of Medicine, Shandong University, No. 44 Wenhua West Road, Jinan, 250012 Shandong China; 2 Department of Biostatistics, School of Public Health, Cheeloo College of Medicine, Shandong University, Jinan, Shandong China; 3 Department of Mailman Public Health, Columbia University, New York, USA; 4 Taian Municipal Hospital, Taian, Shandong China; 5 National Administration of Health Data, No.77 Yuhan Road, Jinan, 250002 Shandong China

**Keywords:** Triglyceride-glucose index, Cardiovascular disease, Trajectory, Longitudinal study

## Abstract

**Background:**

The triglyceride-glucose (TyG) index is regarded as a sophisticated surrogate biomarker for insulin resistance, offering a refined means for evaluating cardiovascular diseases (CVDs). However, prospective cohort studies have not simultaneously conducted baseline and multi-timepoint trajectory assessments of the TyG index in relation to CVDs and their subtypes in elderly participants.

**Methods:**

After excluding data deficiencies and conditions that could influence the research outcomes, this study ultimately incorporated a cohort of 20,185 participants, with data chronicles extending from 2016 to 2022. The TyG index was calculated as Ln [fasting triglyceride (mg/dL) × fasting glucose (mg/dL)/2]. Latent Class Trajectory Model (LCTM) was used to assess the change trends of the TyG index over multiple time points. Utilizing the Cox proportional-hazards models, we assessed the relationship between the baseline quartiles of the TyG index and various trajectories with CVDs and subtypes.

**Results:**

During the mean follow-up time of 4.25 years, 11,099 patients experienced new CVDs in the elderly population. After stratifying by baseline TyG quartiles, the higher TyG level was associated with an increased risk of CVDs; the aHR and 95% CI for the highest quartile group were 1.28 (1.19–1.39). Five trajectory patterns were identified by the LCTM model. The low gradual increase group as the reference, the medium stable group, and the high gradual increase group exhibited an elevated risk of CVDs onset, aHR and 95%CIs were 1.17 (1.10–1.25) and 1.25 (1.15–1.35). Similar results were observed between the trajectories of the TyG index with subtypes of CVDs.

**Conclusion:**

Participants with high levels of baseline TyG index and medium stable or high gradual increase trajectories were associated with an elevated risk of developing CVDs in elderly populations.

**Supplementary Information:**

The online version contains supplementary material available at 10.1186/s12933-023-02100-2.

## Background

Cardiovascular diseases (CVDs) have gradually become the most common cause of death in non-communicable diseases [1–5]. With the acceleration of the aging process, the number of people suffering from cardiovascular diseases has risen sharply, and the prevalence of diseases, especially in the elderly, is more pronounced [6]. The occurrence of CVDs is associated with a variety of risk factors, such as patients with rheumatoid arthritis, systemic lupus erythematosus, depression, etc [7–9]. Nonetheless, there remains a dearth of scientifically robust and straightforward indicators to alert to the imminent risk of CVDs effectively and sensitively [10].

Insulin resistance (IR), a pathological condition where cells fail to respond normally to the hormone insulin, has been shown to have a notable correlation with cardiovascular diseases (CVDs). The relationship underscores the crucial role of metabolic disturbances in the onset and progression of cardiovascular lesions [10, 11]. It not only amplifies the risk associated with traditional CVD factors like dyslipidemia, diabetes, and obesity, but also elevates the CVD risk through alterations in non-traditional factors, such as microalbuminuria [12]. However, in clinical scenarios, assessing IR can be a complex task, and the gold standard approach is both time-intensive and cumbersome, and the homeostasis model assessment for IR has its restrictions [13]. The triglyceride-glucose (TyG) index is an economical and effective new index to evaluate IR [14], which is considered a dependable surrogate marker of IR [15]. To the best of our knowledge, according to the results of cross-sectional studies, the TyG index may be an important marker for assessing CVDs or CVDs factors [16]. However, these studies lack prospective exploration of the TyG index, especially in the elderly population [17]. At the same time, most of the previous studies measured the TyG index at a single time [18], and the impact of the fluctuation and change trajectory of the TyG index on the risk of CVDs in the elderly is unknown.

Based on the above, we aim to explore further and validate the potential correlation between the TyG index and the risk of CVDs in the elderly population. To this end, we have conducted a large-scale prospective cohort study based on an elderly population, including individuals aged 60 and above, with their TyG index measured at least three times from 2016 to 2022. The purpose of the study is to investigate the relationships between the different levels of the TyG index baseline and various trajectories with the onset of CVDs and their subtypes. The outcomes of this research will provide valuable insights for shaping public health policies and clinical practices aimed at the elderly.

## Method

### Study design and population

Data for this study were derived from the Cheeloo Lifespan Electronic Health Research Data-library (Cheeloo LEAD) using the cluster random sampling method to extract data from 5 million people in Shandong Province, China. Cheeloo LEAD establishes a longitudinal cohort based on the National Research Institute’s Data Collection Convergence Common Data Model (RCDM) and Scientific Data Common Data Model (SCDM) and contains information such as health records of residents throughout the life cycle, electronic medical records, health checkups, disease monitoring, medical insurance records, and cause of death monitoring.

**Fig. 1:**
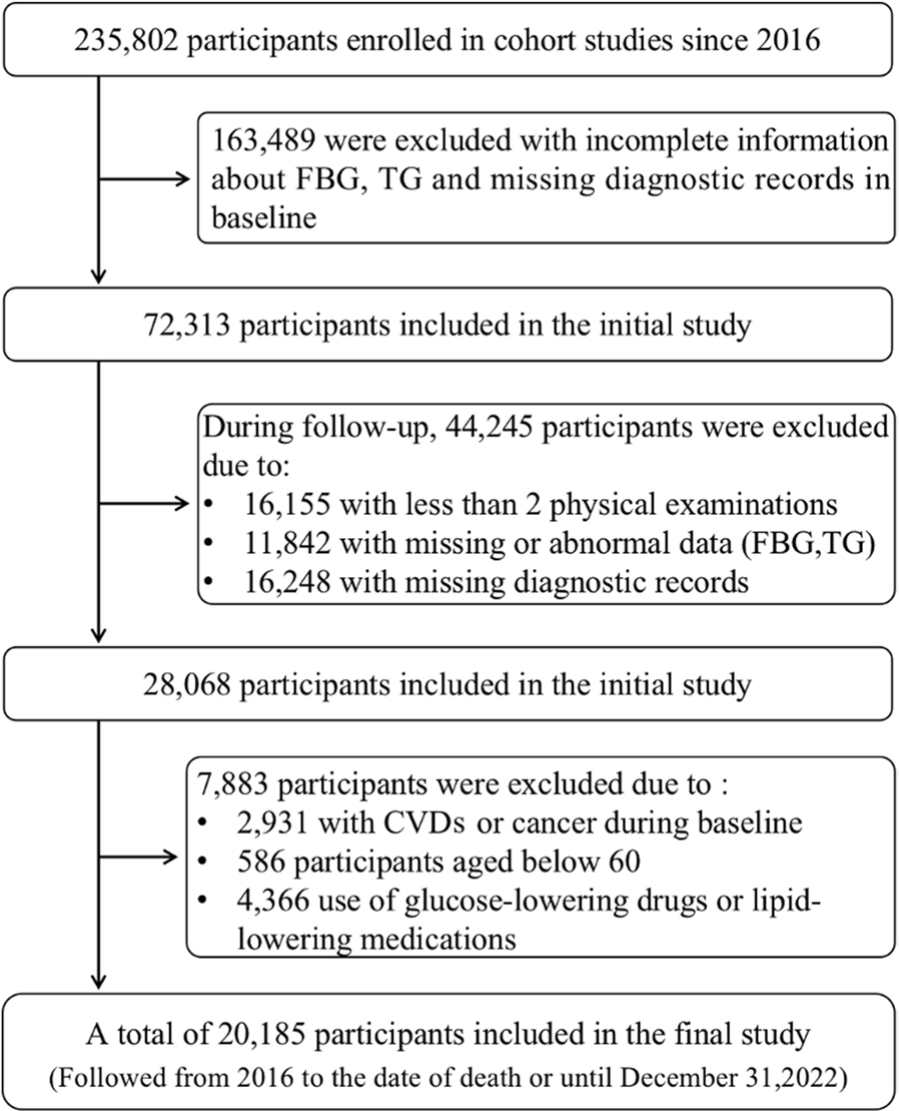
Flowchart of the study participants selection. FBG, fasting blood glucose; TG, triglyceride; CVDs, cardiovascular diseases

Participants were recruited from Cheeloo LEAD, and follow-up information was obtained from 2016 to 2020. A total of 20,185 participants were included in the final longitudinal study after excluding participants with incomplete information about fasting blood glucose (FBG), triglycerides (TG) and missing diagnostic records in baseline(N = 163,489), less than 2 times physical examinations(N = 16,155), missing or abnormal data about FBG or TG and missing diagnostic records during follow-up(N = 28,090), with CVDs or cancer during baseline or age < 60 or using glucose-lowering drugs or lipid-lowering medications(N = 7883) (Fig. 1). All participants signed the informed consent. The study was conducted in accordance with the Helsinki Declaration and approved by the Ethical Committees of the Shandong University School of Public Health (Ethics Number: LL20230904).

### Data collection

In this study, all clinical examination and resident health record information were collected by trained healthcare workers. According to the unique ID code of the individual, data information such as physical examination records, medical records, and death records is matched. All data collection projects are carried out in accordance with standardized procedures. Dietary preferences for salt and oil, as defined by the Dietary Guidelines for Chinese Residents, are characterized by a daily dietary salt intake of more than 5 g (indicating salt craving) and a daily cooking oil intake exceeding 25 g (indicating oil craving) among participants [19].

The resident health record information included items on participant age, sex, education level, and personal habits (smoking, drinking, and diet). Education level was categorized as “Below high school”, “High school”, and “Above high school”. The habit of smoking and alcohol intake were classified as “Current smoking”, “Current non-smoking”, “Current drinking” and “Current non-drinking”. The dietary habits of subjects were assessed by whether they were addicted to salt and oil.

Clinical examinations were performed by experienced nurses and included measurements of height, weight, and blood pressure. Body mass index (BMI) was calculated as weight (kg)/height (m^2^). Hypertension was defined as systolic blood pressure (SBP) ≥ 140 mmHg, diastolic blood pressure (DBP) ≥ 90 mmHg, and the use of any antihypertensive medications. Diabetes was defined as FBG ≥ 7.0 mmol/L or using antidiabetic medication. Dyslipidemia was defined as any of the following: use of lipid-lowering medications, low-density lipoprotein cholesterol (LDL-C) ≥ 4.1 mmol/L, high-density lipoprotein cholesterol (HDL-C) < 1.0 mmol/L, total cholesterol (TC) ≥ 6.2 mmol/L, or triglyceride (TG) ≥ 2.3 mmol/L. And if the history of disease diagnosis was screened in the physical examination or medical consultation data, also defined as having hypertension, dyslipidemia, or diabetes.

### Assessment of TyG index

The TyG index was a composited value composed of TG and FBG levels, calculated as TyG index = ln [TG (mg/dl) × FBG (mg/dl)/2] [14]. The Baseline TyG index was calculated by TG and FBG in baseline (2016). The mean TyG index was calculated as the average value of the TyG index from 2016 to 2022. The TyG index trajectory groups were categorized by the Latent Class Trajectory Model (LCTM). Participants were classified into 5 trajectory groups according to their changes in the TyG index.

### Outcomes

The primary outcome was incident (new-onset) CVDs, including coronary heart disease (CHD), cerebrovascular disease (CVD), peripheral arterial disease (PAD), rheumatic heart disease (RHD), deep vein thrombosis and pulmonary embolism (VTE). All outcomes data in this study were obtained from audited clinical diagnosis records, and professional clinicians make disease diagnoses through imaging, ultrasound, etc. All CVDs events are defined according to the World Health Organization classification. ICD codes from the tenth version were used to identify the diagnosis of CHD (ICD-10 code I21-I25), CVD (ICD-10 code I60-I69), PAD (ICD-10 code I70-I79), RHD (ICD-10 code I01-I02 or I05-I09), VTE (ICD-10 code I26-I28).

### Statistical analysis

The normality of datasets was tested using the Kolmogorov–Smirnov test method. Means and standard deviations (mean ± SD) were used for summarized continuous variables, and percentages and frequencies (N, %) were applied for dichotomized and categorical variables. Variables with a non-normal distribution are represented using the median and interquartile range (IQR) method. The values of the baseline TyG index were ranked and divided into quartiles, the first quartile (Q1) having the fewest and the fourth (Q4) the greatest. The differences between continuous characteristics of TyG quantile groups were assessed by one-way ANOVA. The analysis of differences between groups for continuous variables with a non-normal distribution was conducted using the Kruskal–Wallis H test method. Categorical variables were analyzed by χ^2^ tests.

In this study, the follow-up duration was measured from the time of baseline records to the last follow-up date, and the CVDs events or survival status at the last follow-up was recorded. The survival curve of different TyG groups was plotted with the Kaplan–Meier method and compared by the Log-rank test. The Cox proportional-hazards models were used to assess the risk of events at different baseline TyG levels over time. Both univariate and multivariate analysis methods were performed. In parallel, univariate and multivariate analyses also employed Cox proportional-hazards models to verify the association between TyG index and CVDs or subtypes of CVDs. Results were reported as hazard ratios (HRs) and 95% confidence intervals (95%CIs), and forest plots were used to visualize the results. The dose–response relationship between TyG and CVDs was modeled with restricted cubic splines (RCS) with 4 knots. LCTM was used to assess the change trends of the TyG index over multiple time points, and based on different trends, baseline variables were grouped and analyzed. Sensitivity analysis adjusted for the influence of the baseline TyG index and TyG trajectory on CVDs and their subtypes was conducted by incorporating the mean TyG index into the fully adjusted Cox proportional-hazards model. Statistical significance was determined as a two-sided *P* value less than 0.05.

All statistical analyses and plotting were performed by the R software(version 4.3.1, packages: tidyverse, broom, survival, survminer, ggplot2, splines, forestploter, gtsummary and lcmm).

## Results

### Baseline characteristics and incidence of CVDs according to the baseline TyG index quartiles

A total of 20,185 elderly participants (mean age of 77 ± 6 years, 58.3% women) were included in our study. Table 1 shows the baseline characteristics based on the quartiles of the TyG index. The average baseline TyG index level of the included population was 8.4 ± 0.6. Compared with other groups, the highest group (Q4) of participants was slightly younger, more likely to be female, and had higher BMI, lower percentage of current smoking and alcohol drinking, education level, and halophilic diet, but higher fat diet. Physical examinations or clinical laboratory tests show considerable variability among different groups. We observed higher levels of SBP, TC, TG, LDL-C, HDL-C, and FBG in the quartile group of TyG index. At the same time, a higher prevalence of hypertension, diabetes and dyslipidemia, especially in CVDs was observed in Q4. The incidence of CHD, CVD, and VTE exhibit substantial variability among individual groups, and the rate of the Q4 group was 22.7%, 28.8%, and 0.9%, respectively. According to the quartile grouping based on TyG baseline levels, there are differences in the incidence of CVD, VTE, and CHD among different groups (*P* < 0.001).

**Table 1:** Baseline characteristics and incidence of CVDs according to the baseline TyG index quartiles

Characteristics	Total	Q1(5.7–7.9)	Q2(7.9–8.4)	Q3(8.4–8.7)	Q4(8.7–14.6)	*P* value
N	20,185	5106	5626	4666	4787	< 0.001
TyG index^a^	8.4 ± 0.6	7.7 ± 0.3	8.2 ± 0.1	8.6 ± 0.1	9.2 ± 0.4	< 0.001
Age,years^a^	76.9 ± 6.3	77.4 ± 6.2	77.2 ± 6.3	76.7 ± 6.3	76.0 ± 6.2	< 0.001
Male, n(%)	8411 (41.7)	2807 (55.0)	2315 (41.1)	1752 (37.5)	1537 (32.1)	< 0.001
BMI, kg/m^2a^	24.7 ± 4.2	23.2 ± 3.3	24.6 ± 3.4	25.3 ± 3.4	26.0 ± 5.9	< 0.001
Current smoking, n(%)	13,916 (68.9)	3824 (74.9)	4158 (73.9)	3141 (67.3)	2793 (58.3)	< 0.001
Current drinking, n(%)	16,768 (83.1)	4956 (97.1)	4931 (87.6)	3671 (78.7)	3210 (67.1)	< 0.001
Education, n(%)						< 0.001
< High school	18,931 (93.8)	4942 (96.8)	5305 (94.3)	4318 (92.5)	4366 (91.2)	
High school	878 (4.3)	82 (1.6)	193 (3.4)	245 (5.3)	358 (7.5)	
> High school	376 (1.9)	82 (1.6)	128 (2.3)	103 (2.2)	63 (1.3)	
Salt craving, n(%)	876 (4.3)	299 (5.9)	242 (4.3)	191 (4.1)	144 (3.0)	< 0.001
Oil craving, n(%)	138 (0.7)	45 (0.9)	24 (0.4)	31 (0.7)	38 (0.8)	0.026
SBP, mmHg^a^	117.2 ± 29.0	115.0 ± 18.5	116.4 ± 25.0	117.5 ± 31.5	120.4 ± 38.3	< 0.001
DBP, mmHg^a^	76.0 ± 15.7	76.6 ± 9.1	76.1 ± 13.3	75.7 ± 17.5	75.3 ± 21.2	< 0.001
TC, mmol/L^b^	5.2 (4.5, 5.9)	4.7 (4.1, 5.4)	5.1 (4.5, 5.8)	5.3 (4.7, 6.0)	5.5 (4.9, 6.3)	< 0.001
LDL-C, mmol/L^b^	2.6 (2.1, 3.1)	2.3 (1.8, 2.9)	2.6 (2.1, 3.1)	2.7 (2.2, 3.2)	2.8 (2.4, 3.3)	< 0.001
HDL-C, mmol/L^b^	1.5 (1.2, 1.7)	1.5 (1.3, 1.7)	1.5 (1.3, 1.7)	1.5 (1.3, 1.7)	1.5 (1.2, 1.7)	< 0.001
FBG, mmol/L^a^	4.4 ± 1.3	3.9 ± 0.4	4.1 ± 0.5	4.4 ± 0.9	5.3 ± 2.3	< 0.001
TG, mmol/L^b^	1.2 (0.9, 1.7)	0.7 (0.6, 0.9)	1.2 (1.0, 1.3)	1.6 (1.3, 1.8)	2.3 (1.8, 3.0)	< 0.001
Hypertension,n(%)	5,521 (27.4)	1,040 (20.4)	1,327 (23.6)	1,335 (28.6)	1,819 (38.0)	< 0.001
Diabetes, n(%)	771 (3.8)	10 (0.2)	39 (0.7)	84 (1.8)	638 (13.3)	< 0.001
Dyslipidemia, n(%)	5,999 (29.7)	683 (13.4)	977 (17.4)	1171 (25.1)	3168 (66.2)	< 0.001
CVDs, n(%)	11,099 (55.0)	2567 (50.3)	3176 (56.5)	2611 (56.0)	2745 (57.3)	< 0.001
RHD, n(%)	60 (0.3)	20 (0.4)	13 (0.2)	12 (0.3)	15 (0.3)	0.447
CHD, n(%)	4170 (20.7)	840 (16.5)	1200 (21.3)	1041 (22.3)	1089 (22.7)	< 0.001
CVD, n(%)	5598 (27.7)	1304 (25.5)	1613 (28.7)	1302 (27.9)	1379 (28.8)	< 0.001
PAD, n(%)	984 (4.9)	285 (5.6)	267 (4.7)	212 (4.5)	220 (4.6)	0.055
VTE, n(%)	287 (1.4)	118 (2.3)	83 (1.5)	44 (0.9)	42 (0.9)	< 0.001

Q1, First Quartile; Q2, Second Quartile; Q3, Third Quartile; Q4, Fourth Quartile; TyG index, triglyceride–glucose index; BMI, body mass index; SBP, systolic blood pressure; DBP, diastolic blood pressure; TC, total cholesterol; HDL-C, high density lipoprotein cholesterol; LDL-C, low density lipoprotein cholesterol; FBG, fasting blood glucose; TG, triglyceride; CVDs, cardiovascular diseases; RHD, rheumatic heart disease; CHD, coronary heart disease; CVD, cerebrovascular disease; PAD, peripheral artery disease; VTE, deep vein thrombosis and pulmonary embolism

^a^Data are mean ± SD

^b^Data are median(IQR)

### Association of baseline TyG index with incident CVDs

**Fig. 2:**
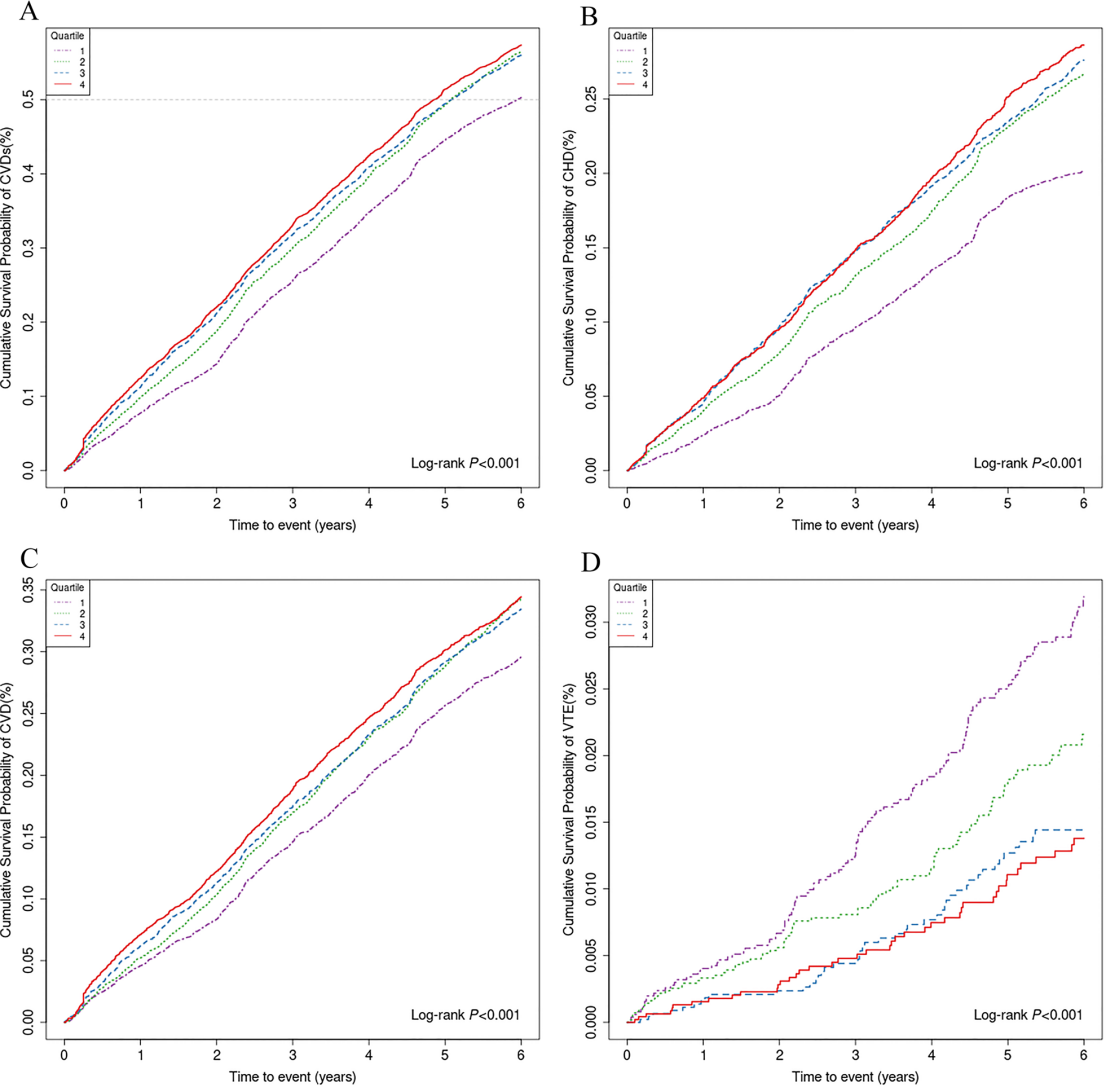
Kaplan‒Meier survival analysis curves for CVDs and subtypes quartiles of baseline TyG index. Incidence of **A** (CVDs), **B** (CHD), **C** (CVD), and **D** (VTE) during follow-up grouped according to the quartiles of baseline TyG index. Cumulative survival probability is presented on the y-axis. Plots use different y-axis scales. TyG index, triglyceride-glucose index; CVDs, cardiovascular diseases; CHD, coronary heart disease; CVD, cerebrovascular disease; VTE, deep vein thrombosis and pulmonary embolism

In the present study of older adults, the mean follow-up time was 4.25 years, and a total of 85,786.25 person-years of follow-up, 11,099 (55.0%) patients experienced new CVDs. Among elderly participants, the incidence of CVDs in Q1-Q4 was 50.3%, 56.5%, 56.0%, and 57.3%, respectively. Kaplan–Meier curve analysis confirmed the significant differences in the incidence of CVDs between quartile groups. The result showed that participants with high levels of the TyG index had a high incidence of CVDs, especially in CVD and CHD, Log-rank *P* < 0.001 (Fig. 2).

**Fig. 3:**
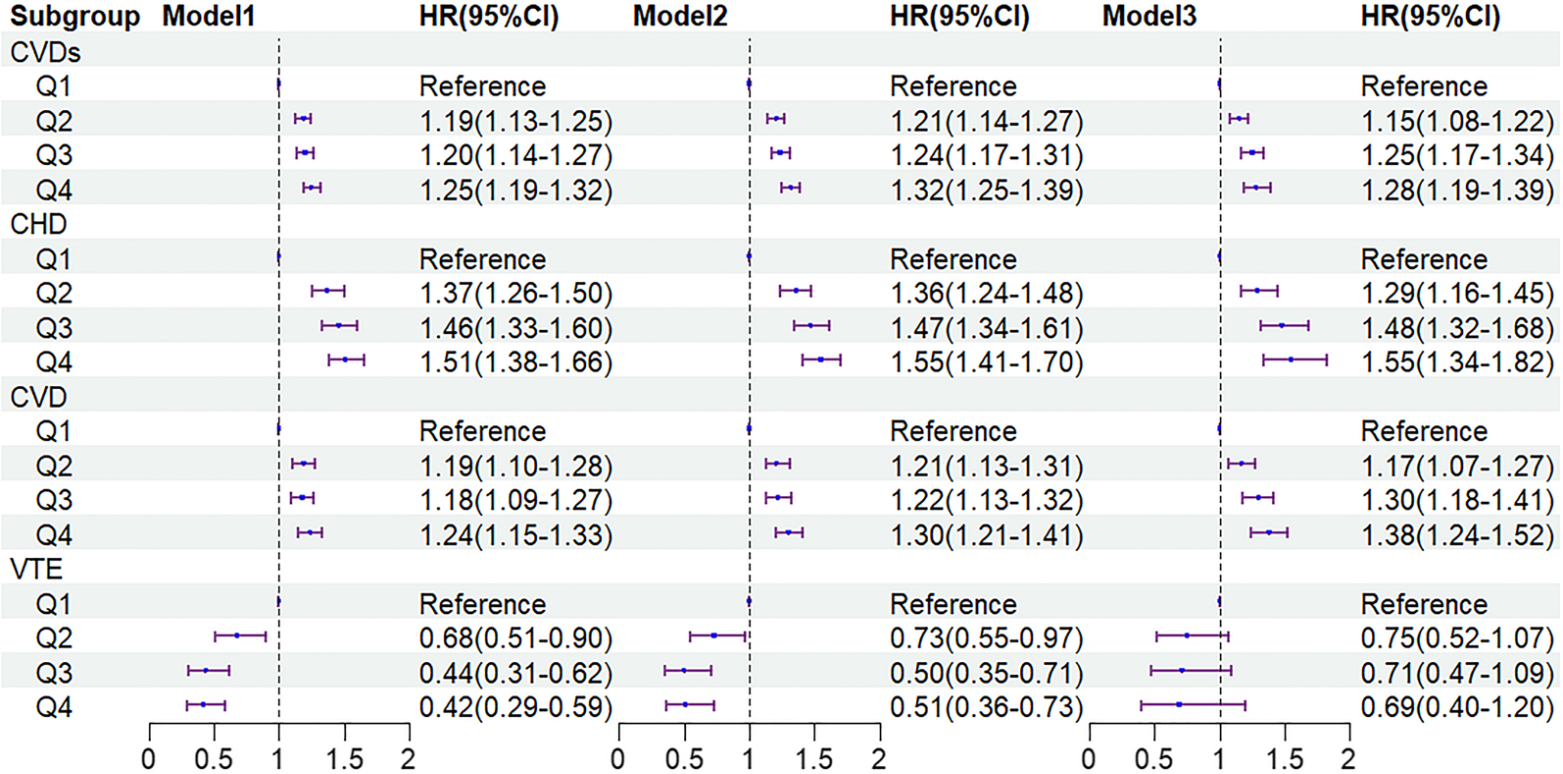
Association between quartiles of the baseline TyG index with the incidence of CVDs. Model 1: unadjusted model; Model 2: adjusted for age sex; Model 3: age, sex, education, BMI, FBG, TG, TC, LDL-C, HDL-C, current smoking, current drinking, oil craving, hypertension, diabetes, dyslipidemia. HR, Hazard Ratio; 95% CI, 95% Confidence Interval; TyG index, triglyceride–glucose index; BMI, body mass index; FBG, fasting blood glucose; TG, triglyceride; TC, total cholesterol; LDL-C, low-density lipoprotein cholesterol; HDL-C, high-density lipoprotein cholesterol; CVDs, cardiovascular diseases; CHD, coronary heart disease; CVD, cerebrovascular disease; VTE, deep vein thrombosis and pulmonary embolism

Figure 3 describes the results of the Cox proportional-hazards regression analysis(Fig. 3). The univariate Cox proportional-hazards regression analysis demonstrated that the highest level of the TyG index group (Q4) was positively associated with the risk of CVDs (HR 1.25; 95% CI 1.19–1.32), compared to the lowest group (Q1). After adjusting for covariates in model 3, significant effects were observed among Q2, Q3, and Q4 groups of TyG index compared with Q1. The aHR increased progressively with a higher level of TyG index; the aHR (95% CI) of different groups versus the lowest quartile were 1.15 (1.08–1.22), 1.25 (1.17–1.34), and 1.28 (1.19–1.39), respectively. Meanwhile, for CHD or CVD event rates, the quantiles of the TyG index were consistent with the overall CVDs results; that is, the higher the level of TyG increases the risk of incident CHD or CVD, aHR, and 95% CI of Q4 vs Q1 in model 3 were 1.55 (1.34–1.82), 1.38 (1.24–1.52), respectively. For the relationship between TyG index quartiles and VTE event rates, differences were less pronounced than for CVD or CHD, *P* > 0.05. After conducting a sensitivity analysis, the results remained consistent (Additional file 2: Table S1).

Dose–response relationships between the TyG index and CVDs were shown by using the restricted cubic spline analysis. The U-shaped curve presented the TyG index level of 8.18 as the reference and non-linear *P* < 0.001(Additional file 1: Fig. S1). For disease subtypes CVD and CHD, the reference value of TyG index was consistent with CVDs.

### Baseline characteristics and incidence of CVDs according to TyG index trajectories

**Fig. 4:**
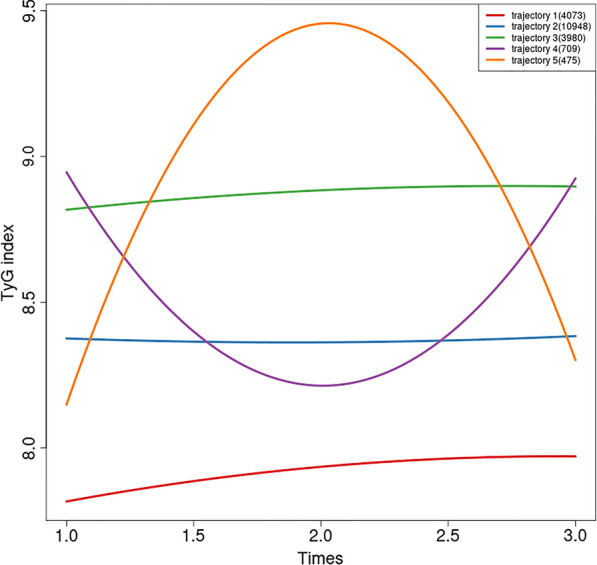
TyG index trajectory groups and percentage of the participants in the group. trajectory1, low gradual increase trajectory; trajectory2, medium stable trajectory; trajectory3, high gradual increase trajectory; trajectory4, increase followed by decrease trajectory; trajectory5, decrease followed by increase trajectory

Through the LCTM model, the TyG index from repeated measurements was divided into trajectories, resulting in five distinct patterns of development, namely the low gradual increase trajectory (trajectory1, N = 4073, 20.18%), the medium stable trajectory (trajectory2, N = 10,948, 54.24%), the high gradual increase trajectory (trajectory3, N = 3980, 19.72%), the increase followed by decrease trajectory (trajectory4, N = 709, 3.51%), and decrease followed by increase trajectory (trajectory5, N = 475, 2.35%) (Fig. 4). The distribution of the baseline demographics and clinical characteristics of the TyG index trajectory was shown in Table 2. Except for the oil craving and the incidence of RHD, which do not show differences among trajectory change groups(*P* > 0.05), statistical differences exist among different trajectory change groups for all other variables(*P* < 0.001). Overall, the trajectory3 and trajectory4 groups had higher levels of TyG index at baseline (9.0 ± 0.4, 9.1 ± 1.2, respectively). Compared to other groups, trajectory3 had higher levels of BMI, SBP, FBG, proportion of hypertension, and diabetes. The trajectory2 and trajectory3 groups exhibit higher incidence rates of CVDs, with the incidence in different TyG trajectory groups being 50.8%, 56.5%, 56.6%, 51.3%, and 48.4%, respectively. For subtypes of CVDs, the incidence of CHD and CVD was higher in the trajectory2 and trajectory3 groups (CHD: 21.7%, 23.0%; CVD:28.7%, 28.2%). The incidence rates of PAD and VTE were higher in trajectory4 and trajectory5, being 7.3% and 7.4% for PAD, 1.8% and 2.1% for VTE, respectively.

**Table 2:** Baseline characteristics and incidence of CVDs according to TyG index trajectories

Characteristics	Total	Trajectory 1	Trajectory 2	Trajectory 3	Trajectory 4	Trajectory 5	*P* value
N	20,185	4073	10,948	3980	709	475	
TyG index^a^	8.4 ± 0.6	7.7 ± 0.3	8.4 ± 0.3	9.0 ± 0.4	9.1 ± 1.2	8.1 ± 0.9	< 0.001
Age,years^a^	76.9 ± 6.3	77.7 ± 6.1	77.1 ± 6.3	75.8 ± 6.1	75.9 ± 6.6	75.6 ± 6.6	< 0.001
Male, n(%)	8,411 (41.7)	2,319 (56.9)	4,321 (39.5)	1,206 (30.3)	333 (47.0)	232 (48.8)	< 0.001
BMI, kg/m^2a^	24.7 ± 4.2	23 ± 3.2	24.9 ± 3.4	26.3 ± 4.9	24.6 ± 9.9	24.0 ± 3.8	< 0.001
Current smoking, n(%)	13,916(68.9)	3125 (76.7)	7867 (71.9)	2185 (54.9)	449 (63.3)	290 (61.1)	< 0.001
Current drinking, n(%)	16,768(83.1)	3933 (96.6)	9302 (85.0)	2503 (62.9)	619 (87.3)	411 (86.5)	< 0.001
Education, n(%)							< 0.001
< High school	18,931 (93.8)	3923 (96.3)	10,287 (94.0)	3595 (90.3)	672 (94.8)	454 (95.6)	
High school	878 (4.3)	78 (1.9)	421 (3.8)	335 (8.4)	27 (3.8)	17 (3.6)	
> High school	376 (1.9)	72 (1.8)	240 (2.2)	50 (1.3)	10 (1.4)	4 (0.8)	
Salt craving, n(%)	876 (4.3)	230 (5.6)	494 (4.5)	97 (2.4)	32 (4.5)	23 (4.8)	< 0.001
Oil craving, n(%)	138 (0.7)	31 (0.8)	63 (0.6)	34 (0.9)	5 (0.7)	5 (1.1)	0.297
SBP, mmHg^a^	117.2 ± 29.0	115.2 ± 18.1	116.6 ± 26.7	121.1 ± 39.9	115.4 ± 36.3	119.4 ± 33.6	< 0.001
DBP, mmHg^a^	76.0 ± 15.7	76.5 ± 8.9	76.1 ± 14.2	75.1 ± 22.4	74.4 ± 20.7	76.6 ± 18.7	< 0.001
TC, mmol/L^b^	5.2 (4.5, 5.9)	4.7 (4.1, 5.3)	5.2 (4.6, 5.9)	5.5 (4.6, 6.2)	5.2 (4.2, 6.2)	4.8 (4.1, 5.6)	< 0.001
LDL-C, mmol/L^b^	2.6 (2.1, 3.1)	2.3 (1.9, 2.8)	2.7 (2.2, 3.1)	2.8 (2.4, 3.3)	2.6 (2.0, 3.1)	2.5 (1.8, 3.0)	< 0.001
HDL-C, mmol/L^b^	1.5 (1.2, 1.7)	1.5 (1.3, 1.7)	1.5 (1.3, 1.7)	1.5 (1.2, 1.7)	1.4 (1.1, 1.7)	1.4 (1.1, 1.7)	< 0.001
FBG, mmol/L^a^	4.4 ± 1.3	3.9 ± 0.3	4.2 ± 0.7	5.3 ± 1.8	5.0 ± 3.9	4.3 ± 2.2	< 0.001
TG, mmol/L^b^	1.2 (0.9, 1.7)	0.7 (0.6, 0.9)	1.3 (1.0, 1.6)	2.0 (1.5, 2.6)	2.8 (1.1, 4.9)	1.0 (0.8, 1.5)	< 0.001
Hypertension, n(%)	5521 (27.4)	760 (18.7)	2764 (25.2)	1611 (40.5)	213 (30.0)	173 (36.4)	< 0.001
Diabetes, n(%)	771 (3.8)	7 (0.2)	151 (1.4)	507 (12.7)	85 (12.0)	21 (4.4)	< 0.001
Dyslipidemia, n(%)	5999 (29.7)	512 (12.6)	2745 (25.1)	2175 (54.6)	459 (64.7)	108 (22.7)	< 0.001
CVDs, n(%)	11,099 (55.0)	2068 (50.8)	6184 (56.5)	2253 (56.6)	364 (51.3)	230 (48.4)	< 0.001
RHD, n(%)	60 (0.3)	17 (0.4)	28 (0.3)	11 (0.3)	2 (0.3)	2 (0.4)	0.570
CHD, n(%)	4170 (20.7)	686 (16.8)	2380 (21.7)	917 (23.0)	123 (17.3)	64 (13.5)	< 0.001
CVD, n(%)	5598 (27.7)	1040 (25.5)	3143 (28.7)	1122 (28.2)	174 (24.5)	119 (25.1)	< 0.001
PAD, n(%)	984 (4.9)	225 (5.5)	506 (4.6)	166 (4.2)	52 (7.3)	35 (7.4)	< 0.001
VTE, n(%)	287 (1.4)	100 (2.5)	127 (1.2)	37 (0.9)	13 (1.8)	10 (2.1)	< 0.001

Trajectory 1, Low gradual increase trajectory; Trajectory 2, Medium stable trajectory; Trajectory 3, High gradual increase trajectory; Trajectory 4, Increase followed by decrease trajectory; Trajectory 5, Decrease followed by increase trajectory; TyG index, triglyceride–glucose index; BMI, body mass index; SBP, systolic blood pressure; DBP, diastolic blood pressure; TC, total cholesterol; HDL-C, high density lipoprotein cholesterol; LDL-C, low density lipoprotein cholesterol; FBG, fasting blood glucose; TG, triglyceride; CVDs, cardiovascular diseases; RHD, rheumatic heart disease; CHD, coronary heart disease; CVD, cerebrovascular disease; PAD, peripheral artery disease; VTE, deep vein thrombosis and pulmonary embolism

^a^Data are mean ± SD

^b^Data are median(IQR)

### Association of TyG index changing trajectory with incident CVDs

**Fig. 5:**
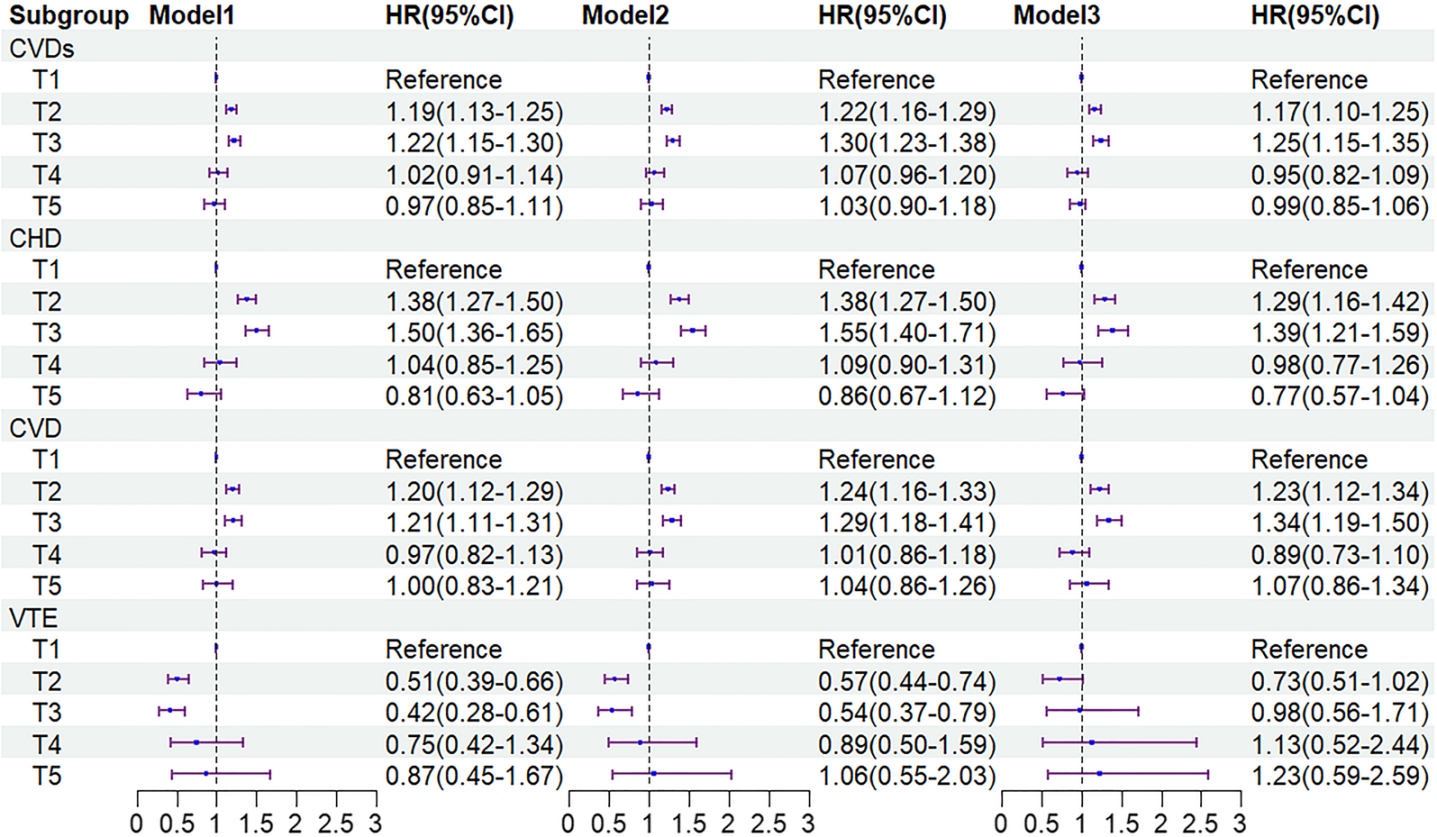
Association between the trajectories of the TyG index and the incidence of CVDs. Model 1: unadjusted model; Model 2: adjusted for age sex; Model 3: age, sex, education, BMI, FBG, TG, TC, LDL-C, HDL-C, current smoking, current drinking, oil craving, hypertension, diabetes, dyslipidemia. HR, Hazard Ratio; 95%CI, 95% Confidence Interval; TyG index, triglyceride–glucose index; BMI, body mass index; FBG, fasting blood glucose; TG, triglyceride; TC, total cholesterol; LDL-C, low-density lipoprotein cholesterol; HDL-C, high-density lipoprotein cholesterol; CVDs, cardiovascular diseases; CHD, coronary heart disease; CVD, cerebrovascular disease; VTE, deep vein thrombosis and pulmonary embolism

The relationships between different TyG index changing trajectories with CVDs, CVD, CHD, and VTE are shown in Fig. 5. Using the trajectory1 group as the reference, in the crude model, both the trajectory2 group and trajectory3 group showed a positive correlation with CVDs; the HRs and 95% CIs were 1.19 (1.13–1.25), 1.22 (1.15–1.30), respectively. After adjusting for multiple factors, the trajectory2 group and trajectory3 group still had 1.17 times (95% CI 1.10–1.25) and 1.25 times (95% CI 1.15–1.35) risk for CVDs. Moreover, similar results were observed in the disease types of CHD and CVD, aHR and 95% CIs were 1.29 (1.16–1.42) and 1.23 (1.12–1.34) in the trajectory2 group, 1.39 (1.21–1.59) and 1.34 (1.19–1.50) in the trajectory3 group. Furthermore, our results found that trajectory4 and trajectory5 showed no statistical significance in relation to the study outcomes, whether considering overall CVDs, CVD, or CHD (*P* > 0.05). After conducting a sensitivity analysis, the results remained consistent (Additional file 3: Table S2). Additionally, while there were differences in the incidence rates of PAD among different trajectory groups, multivariable regression analysis found that the trajectories of the TyG index change were not associated with PAD (*P* > 0.05).

## Discussion

Among the 20,185 elderly participants followed up from 2016 to 2022, significant relationships between different quartiles of the baseline TyG index and varying trajectories with the occurrence of CVDs were observed in the cohort study. Furthermore, the effectiveness of the TyG index on CVDs was still significant despite the adjustment of multiple cardiovascular risk factors. Meanwhile, regarding various CVDs, the TyG index has a similar positive correlation in assessing the risk of CVD, CHD, and VTE onset. These findings revealed the early warning value of TyG index levels and their change patterns for cardio-cerebral events in the elderly.

The result of our current study revealed that compared with a low baseline TyG index, higher levels of TyG index could increase the risk of overall CVDs by approximately 20–30% in the elderly population. Moreover, the negative impact of TyG index on CHD was even greater, with a risk increase ranging from 30% to 80%, and the highest impact on the incidence of CVD has also reached 1.5 times. Our findings suggest that the baseline TyG index could be used to identify individuals at risk of developing kinds of CVDs, and if the TyG index exceeds 8.18, it requires individual attention. Previous studies have also found a positive correlation between TyG and CVDs [3, 20–22], which was consistent with the conclusion of our study. In a longitudinal cohort of 5014 people followed for ten years, the study found that the TyG index can reflect IR, and high levels of the TyG index increased the risk of cardiovascular events by 1.3 times [21]. We confirmed the findings in a larger population and analyzed different cardiovascular diseases. Another recent study indicated that a high level of TyG index may be associated with an increased incidence of coronary artery disease, myocardial infarction, and composite cardiovascular disease (HR: 2.01, 1.36, 1.46, respectively) [22]. The results of these studies are almost consistent with our findings, both proving the effectiveness of the TyG index on cardiovascular events. Among other studies, the restricted cubic spline curves indicated that TyG index between 8 and 9 could be the reference for assessing diseases, such as stroke in cancer survivorship populations and albuminuria among hypertensive participants [23, 24]. These findings are in general agreement with our conclusions.

In our study, we found that elderly individuals with consistently high levels of TyG index, compared to those with low levels, have a higher risk in terms of overall CVDs, CVD, and CHD. In a previous study, the medium stable trajectory of the TyG index was associated with carotid atherosclerosis progression [25]. However, no relationship with carotid atherosclerosis progression was observed in the highly stable group. This conclusion does not entirely align with the findings of our study, which identified that the high gradual increase group’s TyG trajectory was associated with a higher risk of CVDs. The inconsistency may be attributed to the larger number of participants observed in the high gradual increase trajectory group in our study. Meanwhile, the risk assessment value of the long-term trajectories of the TyG index has also been observed to be related to ischemic stroke in the hypertensive population [26]. These studies, as well as our own, have provided evidence of the value of the long-term trajectories of the TyG index in predicting the onset of diseases of the elderly.

CVDs are a leading cause of mortality, with 19 million people worldwide having died from CVDs in 2019 [27]. Age-related increases in disease incidence and metabolic decline are linked to insulin resistance (IR), a core mechanism of metabolic syndrome contributing to cardiovascular events [28, 29]. IR was a significant risk factor for CVDs, linking obesity with type 2 diabetes and CVDs [30]. IR is a significant contributor to the development of diabetes, promoting pathological changes like accelerated atherosclerosis, which is essential in CVD development [31, 32]. At the same time, IR also plays a crucial role in the connection between dyslipidemia and CVDs. IR in adipocytes leads to increased fatty acid release into the bloodstream. This elevation in free fatty acids boosts LDL production in the liver, causing hypertriglyceridemia. High triglyceride levels further increase free fatty acid flow from adipose to non-adipose tissues. Such dysregulation in fatty acid metabolism is crucial in developing endothelial dysfunction, influencing CVDs progression [33, 34]. Studies in recent years have shown TyG index was a surrogate marker of IR [14, 35]. FBG and TG, components of the TyG index, are key indicators of lipid and glucose metabolism. The TyG index could be considered an important indicator for CVDs progression due to its involvement in multiple regulatory mechanisms.

This study has several strengths. First of all, the main strengths of this study are the large size of the elderly cohort and the prospective design. Secondly, to the best of our knowledge, this study is the pioneering investigation into the associations between baseline levels and change trajectories of the TyG index with the incidence of CVDs and their subtypes in the elderly population. Additionally, the TyG index data were measured repeatedly during the follow-up process, and outcome events were collected to explore the causal relationship. At the same time, we adjusted multiple possible risk factors for CVDs to reduce the impact of confounding factors on the outcome.

Despite the strengths, we recognize that there are several limitations to this study. First, our study was an observational study design, lacked randomization and intervention, and we could not eliminate all unmeasured confounding factors. Due to the nature of the observations, we may not be able to draw causal conclusions and will need to intervene to verify causal conclusions in future studies. At the same time, the average age of our study population was relatively older, so the extrapolation of the conclusions of this study may be subject to some limitations.

## Conclusion

In the prospective cohort study in the elderly population, we demonstrated that higher baseline TyG index levels, medium stable, and high gradual increase trajectories are independent risk factors for CVDs, CVD, and CHD. Our findings support the contribution of either one or multiple TyG index measurements to developing CVDs and their subtypes. Therefore, increased vigilance in monitoring the TyG index is essential to mitigate the risk of CVDs.

### Supplementary Information

**Additional file 1: Figure S1.** Restricted cubic spline plots for associations of baseline TyG index levels with CVDs and subtypes. HRs and 95%CIs for A(CVDs), B(CHD), and C(CVD) based on restricted cubic splines for baseline TyG index; HRs and 95%CIs were calculated using Cox proportional-hazards models after adjustment for age. HRs, Hazard Ratios; 95% CIs, 95% Confidence Intervals; CVDs, cardiovascular diseases; CHD, coronary heart disease; CVD, cerebrovascular disease.

**Additional file 2: Table S1.** Sensitivity analysis of the association between the baseline TyG index and CVDs. HR, Hazard Ratio; 95%CI, 95% Confidence Interval; Q1, First Quartile; Q2, Second Quartile; Q3, Third Quartile; Q4, Fourth Quartile; CVDs, cardiovascular diseases; CHD, coronary heart disease; CVD, cerebrovascular disease; VTE, deep vein thrombosis and pulmonary embolism.

**Additional file 3: Table S2.** Sensitivity analysis of the association between the trajectories of the TyG index and CVDs. HR, Hazard Ratio; 95%CI, 95% Confidence Interval; Trajectory1, low gradual increase trajectory; Trajectory2, medium stable trajectory; Trajectory3, high gradual increase trajectory; Trajectory4, increase followed by decrease trajectory; Trajectory5, decrease followed by increase trajectory; CVDs, cardiovascular diseases; CHD, coronary heart disease; CVD, cerebrovascular disease; VTE, deep vein thrombosis and pulmonary embolism.

## Data Availability

The datasets produced in this study are available upon reasonable request by reaching out to the corresponding author.

## Abbreviations

CVDs: Cardiovascular diseases
IR: Insulin resistance
TyG: Triglyceride-glucose
Cheeloo LEAD: Cheeloo Lifespan Electronic Health Research Data-library
RCDM: National Research Institute’s Data Collection Convergence Common Data Model
SCDM: Scientific Data Common Data Model
FBG: Fasting blood glucose
TG: Triglyceride
BMI: Body mass index
SBP: Systolic blood pressure
DBP: Diastolic blood pressure
LDL-C: Low-density lipoprotein cholesterol
HDL-C: High-density lipoprotein cholesterol
TC: Total cholesterol
CHD: Coronary heart disease
CVD: Cerebrovascular disease
PAD: Peripheral arterial disease
RHD: Rheumatic heart disease
VTE: Deep vein thrombosis and pulmonary embolism
HR: Hazard ratio
95%CI: 95% Confidence interval
RCS: Restricted cubic splines
trajectory1: Low gradual increase trajectory
trajectory2: Medium stable trajectory
trajectory3: High gradual increase trajectory
trajectory4: Increase followed by decrease trajectory
trajectory5: Decrease followed by increase trajectory
