# Supplementary figures and images for: Relationship between triglyceride-glucose index baselines and trajectories with incident cardiovascular diseases in the elderly population
Source: Cardiovasc Diabetol. 2024 Jan 3;23:6. doi: 10.1186/s12933-023-02100-2 (PMC10765625; doi:10.1186/s12933-023-02100-2)

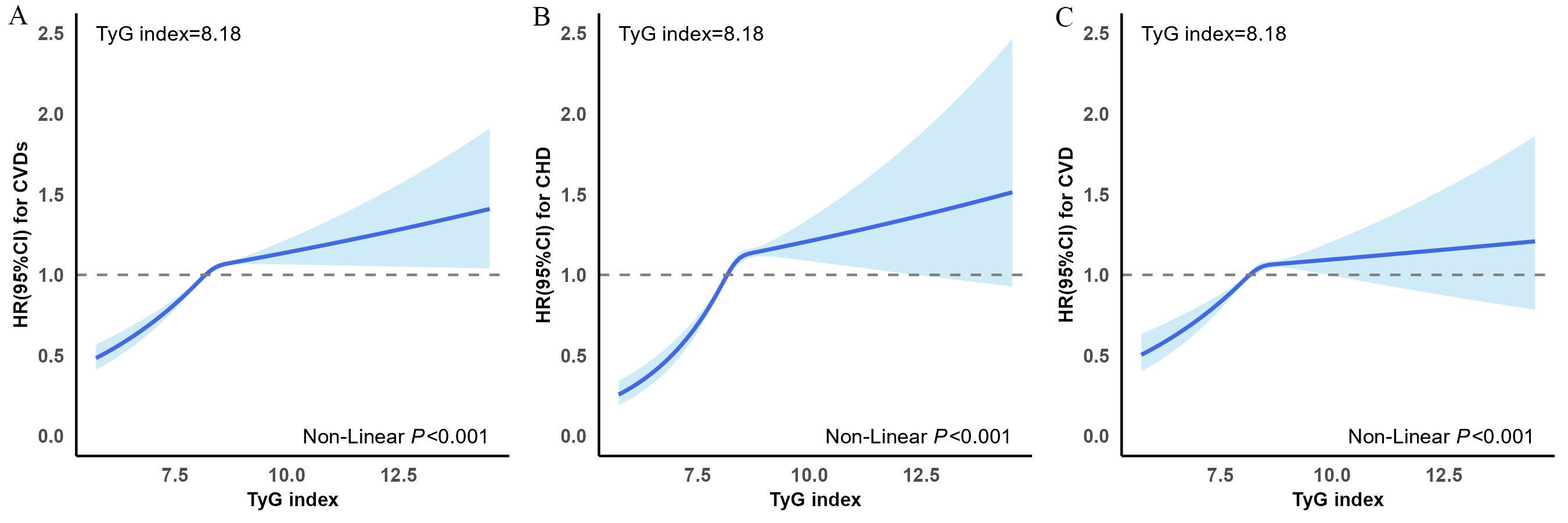

Supplement: Supplementary file 1 — Additional file 1: Figure S1. Restricted cubic spline plots for associations of baseline TyG index levels with CVDs and subtypes. HRs and 95%CIs for A(CVDs), B(CHD), and C(CVD) based on restricted cubic splines for baseline TyG index; HRs and 95%CIs were calculated using Cox proportional-hazards models after adjustment for age. HRs, Hazard Ratios; 95% CIs, 95% Confidence Intervals; CVDs, cardiovascular diseases; CHD, coronary heart disease; CVD, cerebrovascular disease. [file 12933_2023_2100_MOESM1_ESM.jpeg]
