# Supplementary material for: Relationship between triglyceride-glucose index baselines and trajectories with incident cardiovascular diseases in the elderly population
Source: Cardiovasc Diabetol. 2024 Jan 3;23:6. doi: 10.1186/s12933-023-02100-2 (PMC10765625; doi:10.1186/s12933-023-02100-2)
Supplement: Supplementary file 2 — Additional file 2: Table S1. Sensitivity analysis of the association between the baseline TyG index and CVDs. HR, Hazard Ratio; 95%CI, 95% Confidence Interval; Q1, First Quartile; Q2, Second Quartile; Q3, Third Quartile; Q4, Fourth Quartile; CVDs, cardiovascular diseases; CHD, coronary heart disease; CVD, cerebrovascular disease; VTE, deep vein thrombosis and pulmonary embolism. [file 12933_2023_2100_MOESM2_ESM.docx]

**Table S1. Sensitivity analysis of the association between the baseline TyG index and CVDs**

|  | HR | 95%CI | *P* |  | HR | 95%CI | *P* |
| --- | --- | --- | --- | --- | --- | --- | --- |
| CVDs |  |  |  | CVD |  |  |  |
| Q1 | 1 |  |  | Q1 | 1 |  |  |
| Q2 | 1.15 | 1.07-1.22 | <0.001 | Q2 | 1.15 | 1.05-1.27 | <0.001 |
| Q3 | 1.25 | 1.15-1.34 | <0.001 | Q3 | 1.26 | 1.14-1.41 | <0.001 |
| Q4 | 1.27 | 1.15-1.39 | <0.001 | Q4 | 1.33 | 1.17-1.52 | <0.001 |
| CHD |  |  |  | VTE |  |  |  |
| Q1 | 1 |  |  | Q1 | 1 |  |  |
| Q2 | 1.29 | 1.16-1.45 | <0.001 | Q2 | 0.80 | 0.55-1.18 | 0.260 |
| Q3 | 1.48 | 1.31-1.68 | <0.001 | Q3 | 0.79 | 0.49-1.27 | 0.336 |
| Q4 | 1.54 | 1.31-1.82 | <0.001 | Q4 | 0.82 | 0.43-1.53 | 0.527 |

HR, Hazard Ratio; 95%CI, 95% Confidence Interval; Q1, First Quartile; Q2, Second Quartile; Q3, Third Quartile; Q4, Fourth Quartile; CVDs, cardiovascular diseases; CHD, coronary heart disease; CVD, cerebrovascular disease; VTE, deep vein thrombosis and pulmonary embolism.
