# Supplementary material for: Relationship between triglyceride-glucose index baselines and trajectories with incident cardiovascular diseases in the elderly population
Source: Cardiovasc Diabetol. 2024 Jan 3;23:6. doi: 10.1186/s12933-023-02100-2 (PMC10765625; doi:10.1186/s12933-023-02100-2)
Supplement: Supplementary file 3 — Additional file 3: Table S2. Sensitivity analysis of the association between the trajectories of the TyG index and CVDs. HR, Hazard Ratio; 95%CI, 95% Confidence Interval; Trajectory1, low gradual increase trajectory; Trajectory2, medium stable trajectory; Trajectory3, high gradual increase trajectory; Trajectory4, increase followed by decrease trajectory; Trajectory5, decrease followed by increase trajectory; CVDs, cardiovascular diseases; CHD, coronary heart disease; CVD, cerebrovascular disease; VTE, deep vein thrombosis and pulmonary embolism. [file 12933_2023_2100_MOESM3_ESM.docx]

**Table S2. Sensitivity analysis of the association between the trajectories of the TyG index and CVDs**

|  | HR | 95%CI | *P* |  | HR | 95%CI | *P* |
| --- | --- | --- | --- | --- | --- | --- | --- |
| CVDs |  |  |  | CVD |  |  |  |
| Trajectory1 | 1 |  |  | Trajectory1 | 1 |  |  |
| Trajectory2 | 1.15 | 1.07-1.23 | <0.001 | Trajectory2 | 1.18 | 1.07-1.30 | <0.001 |
| Trajectory3 | 1.20 | 1.08-1.33 | <0.001 | Trajectory3 | 1.23 | 1.05-1.43 | <0.001 |
| Trajectory4 | 0.93 | 0.80-1.07 | 0.311 | Trajectory4 | 0.85 | 0.69-1.06 | 0.151 |
| Trajectory5 | 0.96 | 0.82-1.14 | 0.658 | Trajectory5 | 1.02 | 0.80-1.28 | 0.901 |
| CHD |  |  |  | VTE |  |  |  |
| Trajectory1 | 1 |  |  | Trajectory1 | 1 |  |  |
| Trajectory2 | 1.26 | 1.12-1.42 | <0.001 | Trajectory2 | 0.85 | 0.57-1.25 | 0.401 |
| Trajectory3 | 1.33 | 1.11-1.59 | 0.002 | Trajectory3 | 1.38 | 0.70-2.72 | 0.358 |
| Trajectory4 | 0.97 | 0.75-1.24 | 0.783 | Trajectory4 | 1.22 | 0.57-2.59 | 0.611 |
| Trajectory5 | 0.75 | 0.55-1.02 | 0.070 | Trajectory5 | 1.49 | 0.69-3.20 | 0.311 |

HR, Hazard Ratio; 95%CI, 95% Confidence Interval; Trajectory1, Low gradual increase trajectory; Trajectory2, Medium stable trajectory; Trajectory3, High gradual increase trajectory; Trajectory4, Increase followed by decrease trajectory; Trajectory5, Decrease follow by increase trajectory; CVDs, cardiovascular diseases; CHD, coronary heart disease; CVD, cerebrovascular disease; VTE, deep vein thrombosis and pulmonary embolism.
